# “You want them to be partners in therapy, but that's tricky when they’re not there”: A qualitative study exploring caregiver involvement across the continuum of care during the early COVID pandemic

**DOI:** 10.1177/02692155231191011

**Published:** 2023-07-30

**Authors:** Marina B Wasilewski, Zara Szigeti, Christine L Sheppard, Jacqueline Minezes, Sander L Hitzig, Amanda L Mayo, Lawrence R Robinson, Maria Lung, Robert Simpson

**Affiliations:** 1 St. John's Rehab Research Program, 282299 Sunnybrook Research Institute, Sunnybrook Health Sciences Centre, Toronto, Ontario, Canada; 2 Department of Occupational Science and Occupational Therapy & Rehabilitation Sciences Institute, Temerty Faculty of Medicine, University of Toronto, Toronto, Ontario, Canada; 3 Rehabilitation Sciences Institute, Temerty Faculty of Medicine, University of Toronto, Toronto, Ontario, Canada; 4 Musculoskeletal/STAR Rehab and Restorative Transitional Unit, St John's Rehab, Sunnybrook Health Sciences Centre, Toronto, Ontario, Canada; 5 Department of Occupational Science and Occupational Therapy & Rehabilitation Sciences Institute, Temerty Faculty of Medicine, University of Toronto, Toronto, Ontario, Canada; 6 Division of Physical Medicine and Rehabilitation, Department of Medicine, Temerty Faculty of Medicine, University of Toronto, Toronto, Canada; 7 Toronto Rehabilitation Institute, University Health Network, Toronto, Ontario, Canada

**Keywords:** Caregiver, COVID-19, rehabilitation, qualitative

## Abstract

**Objective:**

Widespread visitor restrictions were implemented during the COVID-19 pandemic at acute and inpatient rehabilitation hospitals. Family caregivers were physically isolated from their loved ones, which challenged engagement in patient care and readiness for their role. Thus, we aimed to explore the involvement of family caregivers in COVID-19 patients as they journeyed across the care continuum during the early phase of the COVID-19 pandemic.

**Design:**

We employed a qualitative descriptive approach.

**Participants:**

We conducted interviews with family caregivers, COVID-19 patients, and healthcare providers between August 2020 and February 2021.

**Setting:**

Participants were recruited from a single hospital network in Toronto, Ontario, Canada. Interviews were recorded and transcribed. Data were analyzed thematically.

**Results:**

A total of 27 participants were interviewed—12 healthcare providers, 10 patients, and 5 family caregivers. Four themes were identified: (a) Caregivers were shut out in acute COVID care, (b) Patient discharge from inpatient rehabilitation was turbulent for caregivers, (c) Caregivers were unprepared to support loved ones in the community, and (d) Patient discharge to home was heavily dependent on caregiver availability.

**Conclusions:**

Visitor restrictions prevent family caregivers from being physically present at patients’ bedside, leading to complex and detrimental impacts such as caregivers feeling that they were not engaged in their loved one's care planning until they were discharged. In turn, discharge to the community was met with several challenges including caregivers feeling underprepared and unsupported to meet their loved one's unique care requirements. This was exacerbated by a lack of community-based resources due to ongoing pandemic restrictions.

## Introduction

COVID-19 (COVID) is a respiratory disease associated with multisystem consequences at the respiratory, neuromuscular, and psychosocial levels, including shortness of breath, muscle aches, delirium, depression, social isolation, and even death.^1^ For most patients, COVID presents in a “mild” form; however, other patients experience more serious consequences that must be addressed with medical and rehabilitative care.^2^ During hospitalization and throughout recovery, patients rely on a family caregiver for emotional support, care coordination, and assistance in the community.^3–5^ Despite the essential role that caregivers play in supporting patients’ recovery, infection prevention, and control rules during the pandemic resulted in widespread “visitor restrictions”^6^ that physically isolated caregivers from their loved ones and left them with limited access to clinical supports and educational opportunities.^4,7^ While visitor restrictions have been recently eased in many locations with the arrival of vaccines and less virulent strains, this is likely to vary over time, by setting, and as new variants of the virus emerge. In turn, it remains important to learn from the early period of the pandemic in order to understand how care practices and family involvement have evolved over time and to be prepared for any future reversion to stricter visitation policies.

While there is a growing body of literature illustrating the experiences of family caregivers to COVID patients, most studies have focused on a particular care setting (e.g., community, hospital),^8–10^ thereby limiting our understanding of how family caregiver involvement, experiences, and needs evolved or changed across the care continuum, throughout patient recovery, and during care transitions and discharges.^11^ As such, the goal of this study was to explore the involvement of family caregivers to COVID patients as they journeyed across the care continuum (i.e., from acute care, to inpatient rehabilitation, to the community).

## Methods

This paper reports on data pertaining to the role and involvement of family caregivers, which was collected as part of a study investigating the implementation and impact of COVID care within a single hospital network comprised of an acute care hospital and a linked inpatient rehabilitation facility based in Toronto, Canada. We employed a qualitative descriptive approach, which entails a concise and descriptively rich analysis that remains true to participants’ own words.^12,13^ Thus, it produces a data-near report that is representative of participants’ views, making it meaningful for key stakeholders and relevant for justifying actionable change.^12,13^

Recruitment took place between March and September 2020 and all interviews were conducted between August 2020 and February 2021. At the point of interview, all patient and caregiver participants had been living in the community for at least 3 months after being discharged from the inpatient rehabilitation COVID zone and were able to reflect on their experiences across the continuum of care (i.e., during acute care, inpatient rehabilitation, community living and at transition points across these settings). Throughout the recruitment and during the majority of the interviewing period, strict visitor policies were in place at the hospital network where the study was conducted and more broadly across the province of Ontario. Between March and May of 2020, patients were not allowed any outside visitors in the hospital. Towards the end of May 2020, the provincial Ministry of Health (MOH) released guidelines allowing only “essential visitors” for children/youth, patients who were at the end of life, and those requiring essential support.^14^ In December 2020, hospitals were directed by the MOH to adjust visitor policies according to local outbreaks where the hospital was located.^14^ Visitor restrictions continued to loosen throughout 2021 in alignment with the province's “Roadmap to Re-Open.”^15^

To gain a more holistic and complete understanding of caregiver involvement in COVID care across the continuum, we sought the perspectives of patients, caregivers, and healthcare providers (HCPs). We recruited HCPs working in the hospital network's acute or rehabilitation COVID unit by emailing HCPs on the hospitals’ COVID unit listservs. To recruit patients, we obtained their contact information from the inpatient rehabilitation hospital's patient database. We specifically recruited patients who were discharged from the COVID zone and had been receiving rehabilitation exclusively for COVID. Patients were eligible to participate if they were 18 years or older, English speaking, and cognitively able to provide consent. We recruited caregivers by asking patients if they had someone who was supporting them while recovering from COVID. Patients would then provide their caregivers’ contact information for us to reach out to them. Caregivers were eligible if they were a friend or family member supporting a patient that met the criteria above and were themselves over 18, English speaking and cognitively able to provide informed consent. Caregivers could participate even if the patient chose note to.

This study was approved by the Research Ethics Board at Sunnybrook Health Sciences Centre. Informed consent was obtained prior to data collection. One trained qualitative researcher (SG) conducted all interviews via telephone or Zoom (see Table 1 for interview guide overview). Data were collected until saturation of ideas was reached. The interviewer and the research team were embedded within the inpatient rehabilitation hospital setting. Interviews ranged from 30 to 80 min were audio-recorded and transcribed. All identifying information was removed from the transcripts, which were uploaded to NVivo^16^ for organization and analysis. Sociodemographic information was collected from patients and family caregivers as well as clinical characteristics for patients. We also collected professional practice information from HCPs (e.g., profession, years of practice, practice setting).

**Table 1.** Semi-Structured interview questions for stakeholders.

Stakeholder group	Interview questions
Patients	Could you tell me a little bit about your COVID-19 care journey? Could you tell me what happened when you arrived at [acute care site]? What was it like receiving care at [acute care site] for COVID-19? Overall, what went well during your stay at [acute care site] and what could have been improved? Can you tell me a little bit about your experience of preparing to leave [acute care site] to go rehabilitation? How prepared did you feel for the transition to rehabilitation? How did the transition go? What was it like receiving care at [rehabilitation facility] for COVID-19? Overall, what went well during your stay at [rehabilitation facility] and what could have been improved? How did you feel about getting ready to go home? How did the discharge to home go? After returning to home, did you feel like you had the necessary information and resources to support your continued recovery from COVID-19? Once you were home, what were your top concerns and needs?
Caregivers	Could you tell me a little bit about you and your loved one's COVID-19 care journey? Could you tell me what happened when your loved one arrived at [acute care site]? What was it like to support your loved one while they were receiving care at [acute care site] for COVID-19? Overall, what went well during your loved one's stay at [acute care site] and what could have been improved? Can you tell me a little bit about your experience as your loved one was preparing to leave [acute care site] to go rehabilitation? How prepared did you feel for your loved one's transition to rehabilitation? How did the transition go? What was it like to support your loved one while they were receiving care at [rehabilitation facility] for COVID-19? Overall, what went well during your loved one's stay at [rehabilitation facility] and what could have been improved? How did you feel about your loved one getting ready to go home? How did the discharge to home go? After your loved one returned to home, did you feel like you had the necessary information and resources to support their continued recovery from COVID-19? Once your loved one was home, what were your top concerns and needs?
HCPs	To start, could you tell me a little about your clinical role and work history? What were your thoughts and expectations when you first heard that a portion of [unit] was going to be converted into a COVID Zone? What were your top concerns, questions, and needs when you learned that you would be working in the COVID zone? How do feel your needs/questions/concerns were addressed by managers, administrators and other senior leaders? For managers/administrators/senior leaders: How did you address the needs/questions/concerns that staff had? What has the actual experience of working in the COVID Zone with COVID patients been like? What are your thoughts on the extent and quality of care that was delivered to COVID patients? Reflecting on your experience with working in the COVID Zone, what do you think the top successes were? Reflecting on your experience with working in the COVID Zone, what do you think the top areas for improvement were?

We used a codebook thematic analysis approach guided by several works of Braun and Clarke.^17–19^ Codebook thematic analysis is characterized by the deconstruction of data into isolated fragments using a codebook that delineates the hierarchical relationship between codes. This is then followed by the reconstruction of data into overarching categories and themes that describe “what's going on” in the data. Two independent researchers (ZS and SG) engaged in an open-coding process to generate first impressions that informed an exhaustive codebook that was systematically applied to all transcripts. The coding process was facilitated by the use of NVivo 2010 qualitative software.^16^ Three additional researchers (CS, RS, and MBW) participated in the subsequent stages of thematic analysis, which entailed comparing and contrasting the coded data, categorizing similar ideas, and then grouping categories into distinct and saturated themes (i.e., themes that did not overlap and had enough data to substantiate them).

Analytic rigor was enhanced by triangulating between multiple individuals throughout analysis (thereby enriching the range of perspectives on codes and themes), using a codebook book for consistency across analysts, and providing representative quotes to support researcher interpretations of the data (i.e., enhancing reliability). We also adhered to the COREQ reporting guidelines.^20^

## Results

We interviewed 27 participants for this study, which included 10 patients, 5 caregivers and 12 HCPs. Patients were all admitted for COVID rehabilitation, with *n* = 5 having a primary pulmonary diagnosis, *n* = 4 having a primary diagnosis of “medically complex infection,” and *n* = 1 having COVID-related stroke as a primary diagnosis. Healthcare providers worked in the acute care setting (*n* = 3) and inpatient rehabilitation (*n* = 9). Healthcare providers were occupational therapists (*n* = 3), managers (*n* = 2), registered nurses (*n* = 2), medical department heads (*n* = 2), collaborative practice leaders (*n* = 2), and a pharmacist (*n* = 1). All HCPs (*n* = 12) reported a graduate-level education. Table 2 outlines additional patient and caregiver characteristics.

**Table 2.** Patient and caregiver sociodemographic characteristics (*n* = 15).

Characteristic (mean, SD)	Patient (*n* = 10)	Caregiver (*n* = 5)
Age in years	62.8 (17.9)	60.2 (4.3)
Length of stay in rehabilitation in days	12.4 (1.8)	NA
Characteristic (*n*, %)	Patient (*n* = 10)	Caregiver (*n* = 5)
Positive COVID status upon admission	4 (40)	NA
Sex		
Male	2 (20)	2 (40)
Female	7 (70)	3 (60)
Did not disclose	1 (10)	-
Birth country		
Canada	3 (30)	3 (60)
China	2 (20)	-
Guyana	1 (10)	-
Nigeria	1 (10)	1 (20)
Philippines	2 (20)	1 (20)
USA	1 (10)	-
Ethnicity		
Black	1 (10)	1 (20)
Chinese	2 (20)	-
Filipino	2 (20)	1 (20)
Indian	1 (10)	-
South Asian	1 (10)	-
White	3 (30)	3 (60)
Marital status		
Married	2 (20)	5 (100)
Widowed	4 (40)	-
Single	2 (20)	-
Common law	1 (10)	-
Did not disclose	1 (10)	-
Education		
Some high school	3 (30)	-
Completed college	3 (30)	2 (40)
Completed university	3 (30)	3 (60)
Graduate program	1 (10)	-
Yearly income (CAD$)		
10,000–19,999	2 (20)	-
20,000–29,999	2 (20)	-
30,000–39,999	2 (20)	1 (20)
40,000–49,999	-	1 (20)
50,000–59,999	-	-
60,000–69,999	1 (10)	2 (40)
Did not disclose	3 (30)	1 (20)

The COVID pandemic changed the way family caregivers were involved in and prepared for supporting patient recovery across the continuum of care. With public health restrictions preventing family caregivers from visiting their loved ones during acute care and in-patient rehabilitation, they were forced to play a more distant role in the care of their loved ones while they were in these care settings. The themes below capture the difficulties of family caregivers being physically excluded from the care journey and their sudden uptake of a supportive role in the community setting.

*Theme 1: Caregivers were shut out in acute COVID care*: Active COVID infection clearly impacted the way that patients’ needs were addressed in acute care. Patients were not able to be with their families in person due to restricted visitation policies, and at most, they were able to videoconference or telephone loved ones. In acute care, caregivers described phone communication as limited and “*Strict. I requested that I communicate with her on Skype, but we will always have the problem acting on getting her Skype access. I was only able to do that, maybe, two or three times the whole time she was there”* (CG07). Since caregivers were limited in their abilities to talk directly to their loved ones, they relied heavily on communicating directly with acute care HCPs to obtain information about their loved one's status, medication updates, and general state of health. Many patients were critically ill, sedated, and unable to communicate with loved ones, highlighting why HCPs relayed patient updates by phone:

“So we know what's going on […] I never felt like I was being rushed off the phone. And if something, some numbers or whatever they were using, if metrics were changing in one way or the other, I was able to see whether things were moving in a positive direction or a negative direction. They were quite forthright with saying that, so I always knew that stuff” (CG04).

Once in rehabilitation, family caregivers described a different story regarding involvement in supporting their loved one's recovery. For example, the nature and topics of conversation changed once in rehabilitation. Communication between family caregivers and HCPs was no longer focused exclusively on patients’ survival and health status and began to evolve towards conversations about “*[the patient's] positive mood and recovery*” (CG07). Other caregivers highlighted how they were more “*in on it*” (CG03) due to HCPs better communicating details about the patient's care directly to family members. Similarly, caregivers described communicating with their loved ones with greater ease when they were in rehabilitation. To address issues of social isolation, patients were provided access to technology by the hospital, thereby facilitating freer communication with their families. Some patients and caregivers also described how they were more connected through “window visits”: “*I was able to see [family] from my window, but unfortunately I couldn’t open my window or have them come and see me indoors*” (PT05).

Despite communication having become more feasible and established in rehabilitation, families were still not able to be fully involved in supporting their loved one because they were physically not able to go into the hospital. This made it “*difficult to be engaged from the get go. You want them also to be partners in the therapy, but that's tricky when they’re not there*” (HCP06), leading to an “*impaired ability to bring families into the building to teach them*” (HCP03). In other words, the protocols that inhibited family visitation also prevented the opportunity for families to practice and prepare for their new caregiving responsibilities, which impacted discharge procedures.

*Theme 2: Patient discharge from inpatient rehabilitation was turbulent for caregivers:* Upon discharge from the inpatient rehabilitation facility, caregivers were immediately thrust into a support role after previously being involved only through a telephone or computer screen. Although HCPs and some caregivers described the discharge process as “*smooth”* (HCP02, CG07), other caregivers described feeling unprepared at discharge since “*there were a lot of concerns I didn’t even think about”* (CG03).

With families still being segregated physically from their loved ones at the point of discharge, much of the discharge planning with family caregivers occurred virtually. Discharge meetings were predominately focused on informing caregivers of what physical equipment the patient may have at home. For example, one caregiver described how “*[REHABILITATION] told me they want her to have a certain bath bench or stool or certain kinds of bars to help her be able to get in and out of the tub*” (CG04) but there was less direction from HCPs about what else caregivers should expect in the community (e.g., how much caregivers would be responsible for, types of supportive tasks they would have to undertake). The lack of communication and clarity was perceived as frustrating by caregivers who wanted to provide care out of a desire to “*do whatever is in the [patient's] best interests*” (CG03). Providing this care, however, was described as difficult without information perceived as necessary by caregivers, such as knowing more about how to support loved ones’ needs (e.g., optimizing recovery, keeping them safe at home). Similarly, caregivers were unclear about what to expect with regard to frequency and duration of rehabilitation in the community, perceiving that their loved ones “*weren’t getting enough physio for what [patient] needs*” (CG03).

Additional challenges that caregivers faced at discharge included fears of contracting COVID, lack of awareness about community-based supports, and uncertainty regarding which services would help most. Further, caregivers expected that their loved one would be “*more self-sufficient*” (CG04) following in-patient rehabilitation and were unprepared for the level of dependency that they were confronted with. Overall, caregiver needs and the support they received were mismatched at a critical juncture in patient care and at a time where standard operating procedures for discharge to the community were severely disrupted.

*Theme 3: Caregivers were unprepared to support loved ones in the community:* Following discharge, the return to home was the first time caregivers saw their loved one in person since contracting COVID. Although described as an “*exciting*” time (PT05, CG07, CG10), caregivers faced extensive difficulties when trying to support home-based recovery for their loved one. The context created by COVID accentuated this challenging role, most notably by rendering home-based services largely unavailable because “[c*ommunity workers] didn’t want to get COVID, plain and simple*” (HCP10). Caregivers highlighted that “*there is only so much I can do for [PATIENT] […] because I also have to care for myself and for my husband too*” (CG04). In the absence of community-based services, caregivers had to assume “extended” roles. This was perceived as shocking for some caregivers, with one stating she did not realize she would have to take “*responsibility for someone's toileting, showering, and complete care*” (CG04).

Caregivers highlighted that support services were disjointed when transitioning from inpatient rehabilitation to the community. Already struggling to actually provide the daily personal support needed by loved ones, caregivers also described a lack of continuity in information about equipment needs. One caregiver frustratingly described that the inpatient rehabilitation facility instructed them to obtain certain equipment which was subsequently replaced by the community-based occupational therapist that did a home assessment. The caregiver “*resented that they had me get things that weren’t used…[because] when the occupational therapist eventually arrived on the scene four months or so after [patient] went home, [the occupational therapist] ended up giving her all new equipment. A new rollator. A new bath chair. We had all of that already*” (CG04). To compound matters, caregivers were further hindered by a general lack of practical information about how to care for someone post-COVID. Another caregiver highlighted that obtaining psychological support for their loved one was difficult and “*now that [patient] is home, I think she’d* benefit from some kind of counseling just to help her understand what she went through […] It wasn’t suggested to us. I’m not sure how exactly to go about it” (CG03). Although not a common experience for all caregivers, some of those in the present study had prior experiences of supporting their loved ones, which influenced their capacity to support patient recovery at home. As stated by CG05, “*if we didn’t have that expertise, that experience of having family members go through the rehabilitation process, then I don’t know what we would have done*.” However, even with prior experiences and knowledge of home-based services, these same caregivers still described difficulties and frustrations supporting their loved one's recovery after being excluded from much of the care journey.

*Theme 4: Patient discharge to home was heavily dependent on caregiver availability:* Patients described difficulties in daily activities and household tasks upon discharge home. For some patients, the situation was precarious in that they “*live alone, if I cannot stand on my feet, then I’m really in trouble. So I really struggled and struggled*” (PT15). One patient explained her apprehension to leave inpatient rehabilitation without caregiver support saying “*if [my daughter] could not come, then I dare not come home […] I would’ve stayed at [REHABILITATION] longer*.” Healthcare providers appeared to corroborate, explaining that they “*weren't able to send [patients without caregiver support] home because they wouldn't get their homecare services, so we had several people on the COVID unit stuck [at REHABILITATION]. They were ready to go home, but because they had COVID, the [HOMECARE ORGANIZATION] wouldn't put in their homecare services, so they would go home without help, so we had to keep them. That was a whole other level of stress for the patients, the fear that we would send them home with no help*” (HCP05). Without access to caregiver support in the community post-discharge, the inpatient rehabilitation setting was described as a “placeholder” for patients that had substantial physical deficits.

## Discussion

This qualitative study explored the experiences of family caregivers, patients, and HCPs with COVID care and recovery across the continuum of care during the early phase of the COVID pandemic (March 2020 – February 2021). Findings underscored that caregivers were not well-integrated into patient care planning early on in the recovery process and that it was not until inpatient rehabilitation that their involvement increased. Though involvement during inpatient rehabilitation was enhanced, family caregivers felt that discharge to home often happened too quickly and without enough time for them to prepare. This difficulty was compounded by a lack of community-based support due to the ongoing restriction in those normally available to patients and families. These restrictions and lack of community-based services rendered caregiver support a necessity for patient discharge to home.

A notable and unique aspect of caregiving in the context of the COVID pandemic was the inability of family caregivers to be physically present during their family member's inpatient stay. This lack of physical presence and integration into patient care planning has been reported to deprive caregivers of instructional and learning opportunities that can equip them for the caregiving role.^21^ This was echoed by caregivers in our study who felt that patients’ discharge to the community happened too quickly and that they lacked applied knowledge about the patient's specialized needs to best support their recovery at home. Educating caregivers throughout the course of a disease and following hospitalization has been shown to increase caregivers’ proficiency and self-efficacy with supportive tasks, management skills, and independence.^22^

Caregivers in our study desired information about their family member's hospitalization (e.g., health status, treatment, and prognosis) and required information to help them support their loved one at home (e.g., equipment, community-based psychosocial supports, information about physical care tasks required). These findings are consistent with other COVID caregiving studies that highlight caregivers want regular, scheduled communication from HCPs while their family member is an inpatient (e.g., updates about clinical progress and recovery) and continuity of support after discharge (e.g., to ask questions about patient safety, hygiene, and recovery).^8^ Families have also emphasized how they appreciate easily accessible and frequent communication.^8,23^ Future research should elucidate how to provide caregivers with informational support, especially during periods of physical separation as the pandemic evolves.

Virtual care has expanded at an unprecedented rate during the COVID pandemic. One of the major applications of virtual and technological innovations has been connecting patients and families during periods of physical separation. Maintaining connection between families and patients and involving families in care plans are key tenets of family-centered care practices.^23^ Telehealth and e-communications also have high utility for involving caregivers in patient care planning, allowing them to become educated and prepared for any forthcoming care responsibilities as well as facilitating post-discharge follow-up and support.^11,24^ However, most virtual care policies and programs that emerged during the pandemic simply encouraged caregivers to “keep in touch” virtually or by phone and to ask their family member's care team to facilitate this.^25^ Given that HCPs had limited training and experience with virtual care prior to the pandemic, there are gaps in their ability to use virtual platforms to provide family-centered care and effectively engage caregivers in shared decision-making.^26^ Future research should explore best practices for enabling family-centered care using virtual modalities across the care continuum, with a focus on the resources, staff support, and training required to optimize its implementation.

Finally, our study clearly showed that caregivers felt ill-prepared and unsupported in their role in providing community-based care. While many of these caregiving challenges are not new, they were certainly exacerbated due to the COVID pandemic. Quarantine and other infection prevention regulations created a unique situation where home care services were largely unavailable at a time when caregivers needed them most. To compound a difficult situation, caregivers’ own broader family support systems were unavailable to them too.^27^ The pandemic also created a situation where early discharge was heightened in order to free up beds. This resulted in an increased transference of care from hospital to home, with family caregivers receiving even more limited training and resources than is encountered during non-pandemic circumstances.^22^ The concerning increase in caregiver burden among caregivers of COVID patients should serve as a “red flag.” If societies are to rely on family care during a crisis like this, then lack of preparation and resourcing is a sure recipe for disaster in this new care landscape. Our study lends support to growing calls for more informative training for family caregivers of COVID patients to enhance their capacity for community-based care.^22^ As mentioned previously, one avenue that has potentially been under-utilized to date for caregiver training is virtual care.

A notable strength of this study is the inclusion of patients, caregivers, and HCPs which enabled us to explore the role and needs of families from multiple stakeholder perspectives. We were also able to describe participant experiences across the continuum of care, which shed light on transition-specific issues and discharge-related challenges. Participants in our study were English-speaking and had mid-to-high socioeconomic status (SES), limiting findings’ transferability to linguistically diverse individuals and those from lower SES. Our study only included individuals that *actually took on* the caregiving role and did not capture the experiences of those who chose not to. Finally, our study was conducted during the early stages of the pandemic and thus the perspectives of stakeholders may change or evolve as policies and procedures are modified based on emerging knowledge of COVID, vaccine availability, and the emergence of new variants.

**Clinical messages**

- Clinicians must prioritize regular communication with family caregivers and ensure availability of ongoing support after discharge.
- Better training for family caregivers of COVID patients is needed to enhance their capacity for community-based care.
- Clinical best practices that enable family-centered care using virtual modalities would enhance cross-continuum support for caregivers.

## References

[1] Chen N Zhou M Dong X , et al. Epidemiological and clinical characteristics of 99 cases of 2019 novel coronavirus pneumonia in Wuhan, China: a descriptive study. Lancet 2020; 395: 507–513. 32007143 10.1016/S0140-6736(20)30211-7 PMC7135076

[2] Rodriguez-Morales AJ Cardona-Ospina JA Gutierrez-Ocampo E , et al. Clinical, laboratory and imaging features of COVID-19: a systematic review and meta-analysis. Travel Med Infect Dis 2020; 34: 101623. 32179124 10.1016/j.tmaid.2020.101623 PMC7102608

[3] Naylor MD Hirschman KB McCauley K . Meeting the transitional care needs of older adults with COVID-19. J Aging Soc Policy 2020; 32: 387–395. 32476586 10.1080/08959420.2020.1773189

[4] Irani E Niyomyart A Hickman RL Jr . Family caregivers’ experiences and changes in caregiving tasks during the COVID-19 pandemic. Clin Nurs Res 2021; 30: 1088–1097. 33998836 10.1177/10547738211014211 PMC8366190

[5] Moss SJ Krewulak KD Stelfox HT , et al. Restricted visitation policies in acute care settings during the COVID-19 pandemic: a scoping review. Crit Care 2021; 25: 47. 34563234 10.1186/s13054-021-03763-7 PMC8465762

[6] Jaswaney R Davis A Cadigan RJ , et al. Hospital policies during COVID-19: an analysis of visitor restrictions. J Public Health Manag Pract 2022; 28: E299–E306. 10.1097/PHH.0000000000001320 33729198

[7] Bergmann M Wagner M . The impact of COVID-19 on informal caregiving and care receiving across Europe during the first phase of the pandemic. Front Public Health 2021; 9: 673874. 34222177 10.3389/fpubh.2021.673874 PMC8242257

[8] Picardi A Miniotti M Leombruni P , et al. A qualitative study regarding COVID-19 inpatient family caregivers’ need for supportive care. Clin Pract Epidemiol Ment Health 2021; 17: 161–169. 35136412 10.2174/1745017902117010161 PMC8719278

[9] Rahimi T Dastyar N Rafati F . Experiences of family caregivers of patients with COVID-19. BMC Fam Pract 2021; 22: 1–10. 34187368 10.1186/s12875-021-01489-7 PMC8241402

[10] Moss SJ Krewulak KD Stelfox HT , et al. Perspectives from designated family caregivers of critically ill adult patients during the COVID-19 pandemic: a qualitative interview study. PLoS One 2022; 17: e0275310. 10.1371/journal.pone.0275310 PMC9514636 36166458

[11] Sumikawa Y Yamamoto-Mitani N . Transitional care during COVID-19 pandemic in Japan: calls for new strategies to integrate traditional approaches with information and communication technologies. Biosci Trends 2021; 15: 55–57. 33627572 10.5582/bst.2021.01056

[12] Sandelowski M . Whatever happened to qualitative description? Res Nurs Health 2000; 23: 334–340. 10940958 10.1002/1098-240x(200008)23:4<334::aid-nur9>3.0.co;2-g

[13] Sandelowski M . What's in a name? Qualitative description revisited. Res Nurs Health 2010; 33: 77–84. 20014004 10.1002/nur.20362

[14] Borden Ladner Gervais LLP. Ontario hospital visitor restrictions during the COVID-19 pandemic: December 2020 update, 2020.

[15] Ontario Health. Toronto Region COVID-19 Hospital Operations Table Recommended Visitor Restrictions with Exceptions for Acute, Rehabilitation And Complex Continuing Care Hospitals, 2021.

[16] NVivo qualitative data analysis software. 10th ed. QSR International Pty Ltd., 2010.

[17] Braun V Clarke V . Using thematic analysis in psychology. Qual Res Psychol 2006; 3: 77–101.

[18] Braun V Clarke V . One size fits all? What counts as quality practice in (reflexive) thematic analysis? Qual Res Psychol 2021; 18: 328–352.

[19] Braun V Clarke V . Reflecting on reflexive thematic analysis. Qual Res Sport Exerc Health 2019; 11: 589–597.

[20] Tong A Sainsbury P Craig J . Consolidated criteria for reporting qualitative research (COREQ): a 32-item checklist for interviews and focus groups. Int J Qual Health Care 2007; 19: 349–357. 17872937 10.1093/intqhc/mzm042

[21] Camicia ME Cournan MC Rye J . COVID-19 and inpatient rehabilitation nursing care: lessons learned and implications for the future. Rehabil Nurs 2021; 46: 187–196. 34009902 10.1097/RNJ.0000000000000337 PMC8270222

[22] Mirzaei A Raesi R Saghari S , et al. Evaluation of family caregiver burden among COVID-19 patients. Open Public Health J 2020; 13: 808–814.

[23] Ashana DC Cox CE . Providing family-centered intensive care unit care without family presence—human connection in the time of COVID-19. JAMA Netw Open 2021; 4: e2113452. 10.1001/jamanetworkopen.2021.13452 34152421

[24] Munshi L Evans G Razak F . The case for relaxing no-visitor policies in hospitals during the ongoing COVID-19 pandemic. CMAJ 2021; 193: E135–E137. 10.1503/cmaj.202636 PMC7954559 33355198

[25] Fiest KM Krewulak KD Hiploylee C , et al. An environmental scan of visitation policies in Canadian intensive care units during the first wave of the COVID-19 pandemic. Can J Anesth 2021; 68: 1474–1484. 34195922 10.1007/s12630-021-02049-4 PMC8244673

[26] Wittenberg E Goldsmith JV Chen C , et al. Opportunities to improve COVID-19 provider communication resources: a systematic review. Patient Educ Couns 2021: 438–451. doi:10.1016/j.pec.2020.12.031 33455825 PMC7831717

[27] Dang S Penney LS Trivedi R , et al. Caring for caregivers during COVID-19. J Am Geriatr Soc 2020; 68: 2197–2201. 10.1111/jgs.16726 PMC7361597 32638348

